# Increase on environmental seasonality through the European Early Pleistocene inferred from dental enamel hypoplasia

**DOI:** 10.1038/s41598-023-42936-y

**Published:** 2023-10-07

**Authors:** Darío Fidalgo, Antonio Rosas, Saverio Bartolini-Lucenti, Jean-Renaud Boisserie, Luca Pandolfi, Bienvenido Martínez-Navarro, Paul Palmqvist, Lorenzo Rook, Joan Madurell-Malapeira

**Affiliations:** 1 Department of Palaeobiology, Museo Nacional de Ciencias Naturales (CSIC), c/José Gutiérrez Abascal 2, 28006 Madrid, Spain; 2 Earth Sciences Department, Paleo[Fab]Lab, Università di Firenze, Via G. La Pira 4, 50121 Florence, Italy; 3 Institut Català de Paleontologia Miquel Crusafont, Universitat Autònoma de Barcelona, Edifici ICTA-ICP, c/ Columnes s/n, Campus de la UAB, 08193 Cerdanyola del Vallès, Spain; 4 Laboratory Paleontology Evolution Paleoecosystems Paleoprimatology (PALEVOPRIM, UMR CNRS 7262), University of Poitiers, Poitiers, France; 5 Dipartimento di Scienze, Università della Basilicata, Viale dell’Ateneo Lucano, 10, 85100 Potenza, Italy; 6 Institut Català de Paleoecologia Humana i Evolució Social (IPHES-CERCA), Zona Educacional 4, Campus Sescelades URV (Edifici W3), 43007 Tarragona, Spain; 7 Area de Prehistoria, Universitat Rovira i Virgili (URV), Avda. Catalunya 35, 43002 Tarragona, Spain; 8 ICREA, Pg. Lluís Companys 23, 08010 Barcelona, Spain; 9 Departamento de Ecología y Geología, Universidad de Málaga, Campus de Teatinos, 29071 Málaga, Spain; 10 Department of Geology, Faculty of Sciences, Universitat Autònoma de Barcelona, Cerdanyola del Vallès, Spain

**Keywords:** Ecology, Evolution, Environmental sciences

## Abstract

An in-depth study of the Early Pleistocene European remains of *Hippopotamus* has allowed the first detailed description of the incidence and types of dental alterations related to palaeopathologies and potentially linked to climatic and environmental factors. The results of a long-term qualitative and quantitative assessment highlight the importance of nutrient deficiencies on the development of dental enamel hypoplasia in *Hippopotamus*. Glacial cyclicity and the resulting changes in humidity and plant community structure conditioned the local environments critical for the survival of this taxon. Two main intervals of putative constrained nutritionally restrictions were detected at ca. 1.8 Ma and ca. 0.86 Ma (i.e., MIS63 and MIS21, respectively). Statistical comparisons show an increase in the frequency of dental hypoplasia between these two chronological periods, thus reinforcing the idea of increased seasonality in the circum-Mediterranean environments during the Early Pleistocene.

## Introduction

### Paleoclimatic background

Throughout the Pliocene and Pleistocene, long-term trends of climatic cooling and increased glacial cycle amplitude are suggestive of significant changes in the dynamics of the Earth climate system^1^. In addition, a sustained trend towards increased aridification and seasonality in Europe throughout the Pleistocene is generally accepted^2^, which forced a progressive transition from tropical-subtropical ecosystems to present-day temperate ones^3,4^. In response to the aforementioned changes, the European large mammal assemblages biodiversity recorded an increase in the frequency of species adapted to more open environments, which was coincident with the first hominin dispersal out of Africa^5–7^. Moreover, a progressive increase in the amplitude of climate oscillations is documented since 1.4 Ma onwards, including a shift from 41-kyr to ca. 100-kyr orbital rhythm, an increase in the long-term average ice volume, and the establishment of a strong asymmetry in glacial ice volume cycles (i.e., ‘*The Early-Middle Pleistocene Transition*’ [EMPT] sensu Head and Gibbard^2^).

### Background on Dental Enamel hypoplasia and *Hippopotamus* paleoecology

Dental Enamel Hypoplasia (DEH) is a disruption in enamel secretion by ameloblasts during amelogenesis, which is linked to physiological stresses^8^. There are three broad categories of DEH according to FDI (Federation Dentaire International^9^) and, according to Hillson^10^ and Hillson and Bond^11^, DEH displays a wide array of expressions, comprising of vertical or horizontal grooves, pits, or broad bands of missing or incomplete enamel. In large mammals, such disturbances are often associated with periods of environmental and/or nutrient stresses, resulting in a metabolic stress that causes this pathological state^8,12^. Many studies of the relationships between DEH and the environmental conditions in which the individuals lived have been published^13–15^. The position, width, and depth of DEH can provide information on the age of occurrence of anomaly, the period of stress event, and the severity of the stressors^16,17^.

Among the large mammals that inhabited Europe during the Pleistocene, the large-sized *Hippopotamus* spp. had several environmental requirements (such as the presence of permanent bodies of water or the abundance of grassland or aquatic macrophytes) making them relevant for climatic studies^18^. Their climatic range has been considered narrow, conditioning the use of their presence as a clear indicator of warm and humid climates^19^. However, preferential diet of individuals of Early Pleistocene European populations of (ca.3200 kg) *Hippopotamus antiquus* is still debated*.* Specifically, the discussion continues between a grazing behaviour similar to the dietary preferences of extant *Hippopotamus amphibius*^20^ and a diet centred on aquatic macrophytes, as suggested by biogeochemical data from the late Early Pleistocene site of Venta Micena^21^.

Despite the specific ecological requirements related to water availability and the characteristic continuous growth of their anterior dentition, DEH in hippopotamuses has been scarcely reported^20,22^ and no comprehensive study has been published so far, especially in relation to changes in the biotic or abiotic factors of the environment. In any case, no comprehensive study of DEH in hippopotamuses or any long-term projection of this phenomenology against changes in the biotic or abiotic environment has been carried out so far.

**Figure 1:**
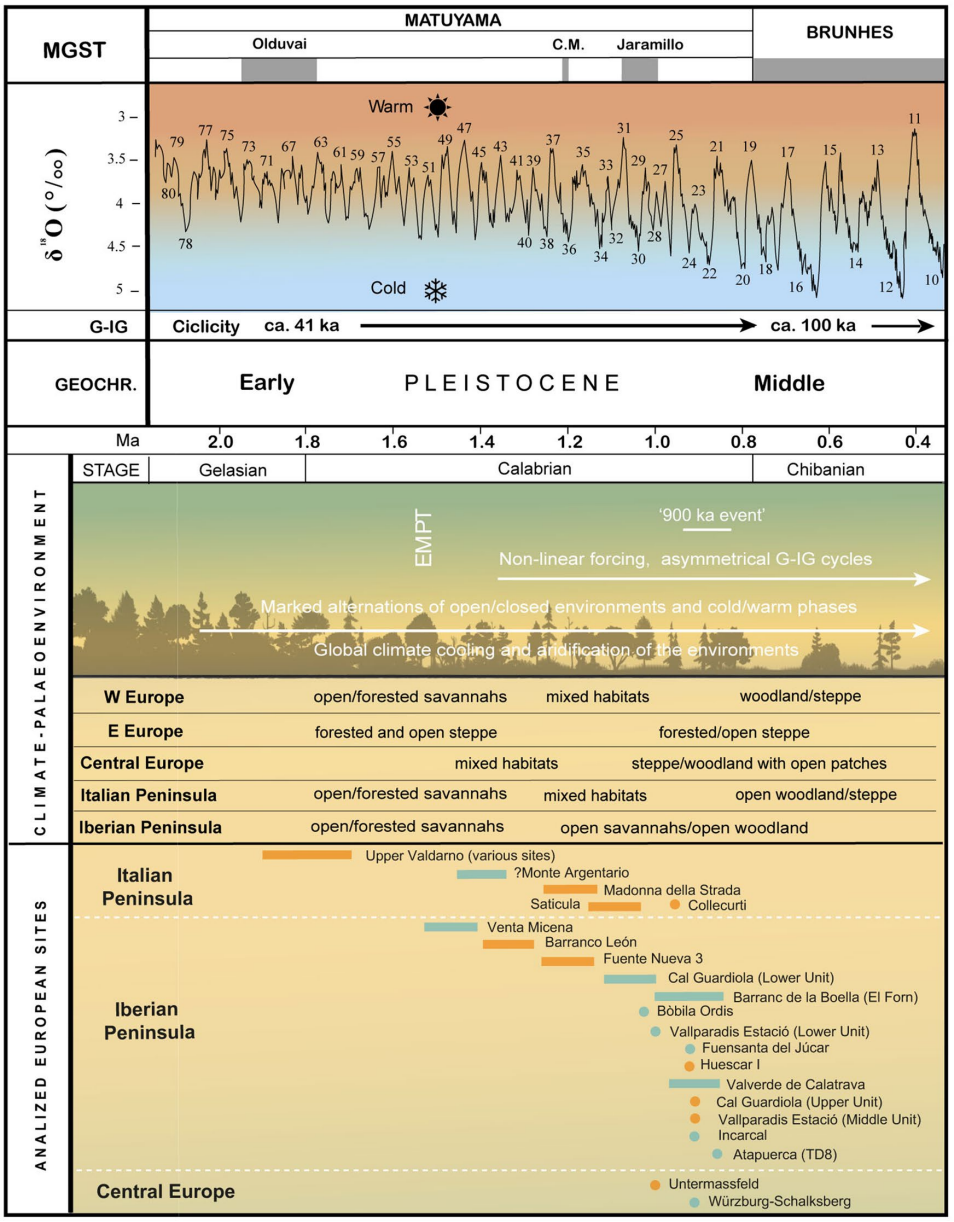
Geographical and chronological location of the samples included in this study. Chronological representation also includes epochs/ages, the marine isotopic curve and the most important climatic events in Europe during the Early Pleistocene. Upper Valdarno comprises specimens from: Upper Valdarno general area (more details unknown), Castelfranco, Cava Bacchi, Figline, Renacci, Vacchereccia, Malpasso and Il Colombaiolo. Above the known chronological ranges of each locality (lines or dots), sites with documented hypoplasia are marked in orange; in blue, sites where it was not detected. Marine Isotopic Stages information from Lisiecki and Raymo^1^. Climatic Events information from Kahlke et al.^42^.

Following the observation of a large representation of DEH in the anterior dentition of the European hippopotamuses (*H. antiquus*) found in several Early Pleistocene layers at different sites in Europe (Fig. 1), this paper evaluates the question of whether this pathological state may be associated with changes in climatic condition that may resulted in nutritional stress.

## Results

### Chronological and geographical distribution of Enamel Hypoplasia

**Figure 2:**
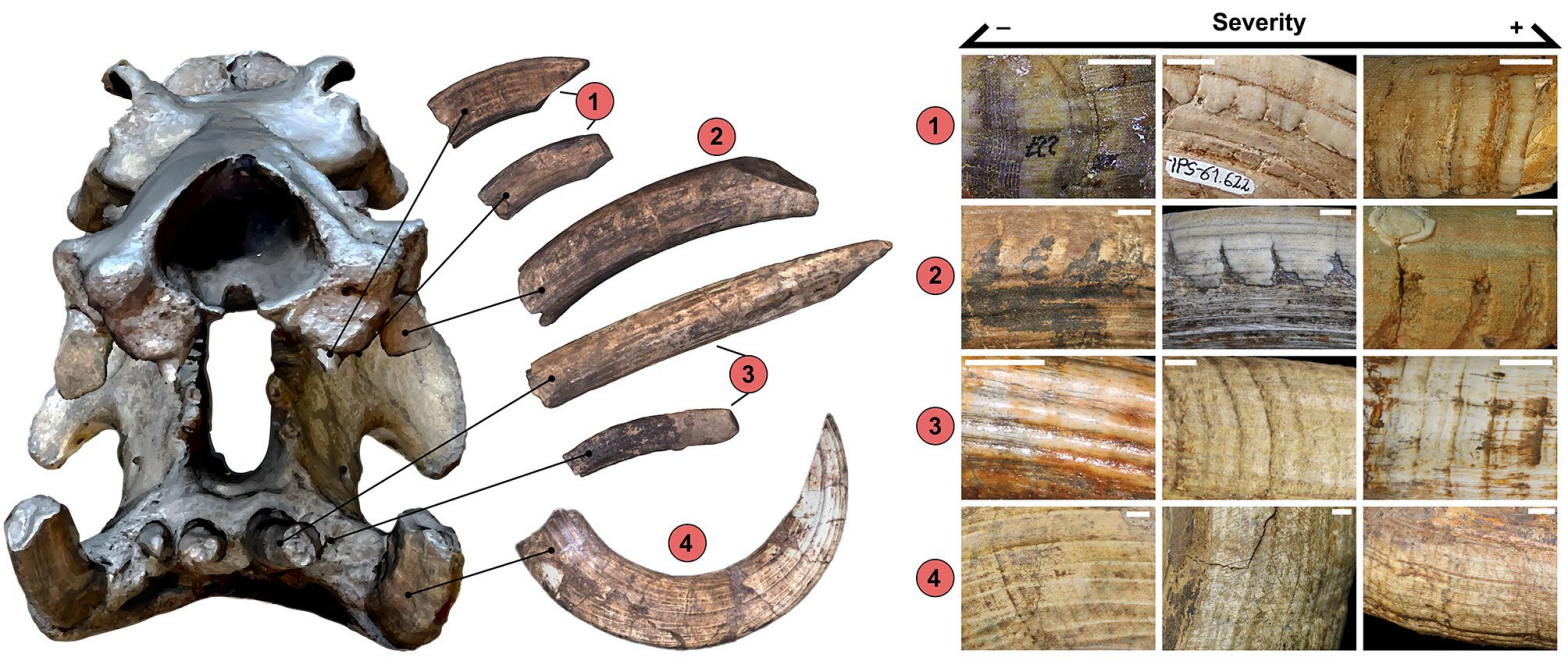
Skeletal elements included in the analysis and the different degrees of severity of Linear Enamel Hypoplasia (LEH) observed in each element. Skull specimen: AC3864. 1: upper incisors, 2: upper canines, 3: lower incisors, 4: lower canines. From minimum to maximum severity of LEH: periodic appearance of lines with change of enamel colouring; onset of enamel loss from the enamel-dentine contact limits; appearance of periodic bands with absence of enamel with more than half of the development of the enamel layer. Scale bar equals 20 mm.

The analysis of the sample of incisors and canines showed 105 pathologies related to the production of dental enamel among the 310 specimens analysed (30 among 74 Minimum Number of Individuals; Table S1). Twelve of the 23 Early Pleistocene sites studied around Southern and Central Europe document specimens with enamel hypoplasia, although with different incidence (Figs. 1, 2 and S1; Table S1). Of these sites, only the samples of Upper Valdarno and Vallparadís Section have enough specimens to be significative (see “Material and methods”).

**Figure 3:**
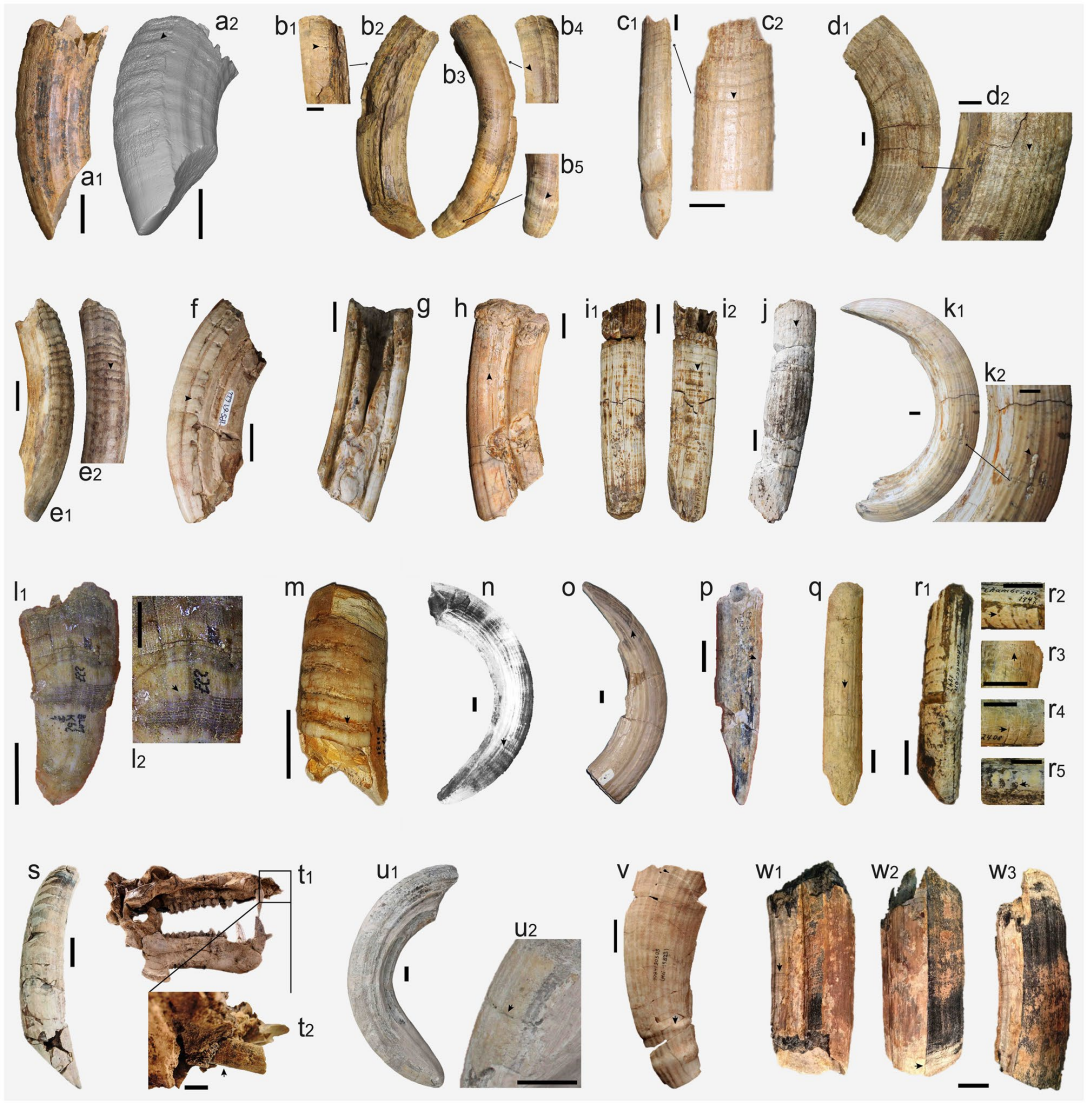
Examples of anterior dentition of *Hippopotamus antiquus* with Dental Enamel Hypoplasia (DEH) observed in the sample evaluated. (**a**): upper incisor with LEH (IGF 790), (**b**): upper canine with LEH (IGF 774), (**c**): lower incisor with LEH (IGF 804) and (**d**): lower canine with LEH (IGF 788) from Upper Valdarno; (**e**): upper incisor with LEH (IPS127144), (**f**): upper incisor with LEH (IPS61622), (**g**): upper canine with malformation (IPS98656), (**h**): upper canine with a traumatic episode and malocclusion (IPS48787), (**i**): lower incisor with LEH (IPS127126), (**j**): lower incisor with LEH (IPS879) and (**k**): lower canine with PEH (IPS127044) from post-Jaramillo Units of Vallparadis Section; (**l**): upper incisor from Barranco León with LEH (BL01-K52-1); (**m**): upper incisor with PIEH (MNCN19365) and (n): lower canine with LEH (Hu-1-105) from Huescar-I^43^; (**o**): lower canine with LEH from Cava Santarelli-Madonna della Strada^44^; (**p**): upper incisor with LEH (FN3-T10-6B) and (**q**): lower canine with LEH (FN303-O93-4) from Fuente Nueva-3; (**r**)1,2 (g.2410), (**r**)3 (g.2407), (**r**)4 (g.2408), (**r**)5 (320 B29): upper incisors with LEH from Chambezon; (**s**): upper canine with LEH and t2,3: upper incisor with LEH preserved in the skull from Collecurti exposed in the Museo Paleontologico Archeologico Serravalle di Chienti; (**u**): lower canine with LEH (IPS14519) from Esparraguera; (**v**): lower canine with LEH from Untermassfeld^20^; (**w**): upper canine with LEH from Saticula^45^. LEH: Linear Enamel Hypoplasia, PEH: Pit Enamel Hypoplasia, PIEH: Plane Enamel Hypoplasia. Scale bar equals 20 mm.

Samples of specimens showing Dental Enamel Hypoplasia are observed essentially in two periods: in the ca. 1.9–1.7 Ma interval at the Upper Valdarno sites and in the ca. 1.0–0.86 Ma interval at the Vallparadís Section. Additionally, five specimens displaying hypoplasia are found in Barranco León (BL01-K50-6; Fig. 3i), Fuente Nueva 3 (FN3-T10-6B, FN303-O93-4; Fig. 3m,n), Cava Santarelli (Madonna della Strada; Fig. 3o) and Saticula (Fig. 3w) in the 1.4–1.2 Ma interval (Table S1; Fig. 1). It is important to consider the possibility of obtaining random (or at least uninterpretable) results in sites where the sample of specimens is < 10. This situation especially affects some of the possible observations in the 1.6–1.0 Ma timeframe (Table S1; Fig. 1).

### Types and incidence of pathologies

Different pathologies such as traumatic episodes (Fig. 3h), malformations (Fig. 3g) or malocclusion (Fig. 3h) coincide with Linear Enamel Hypoplasia (LEH) (Fig. 2), Pit Enamel Hypoplasia (PEH) (Fig. 3k) and Plane Enamel Hypoplasia (PlEH) (Fig. 3m). Clear banding patterns with some enamel waviness, lacking pigmentation and episodes of enamel loss are observed mainly in canines and upper incisors with LEH and PlEH (Fig. 3a–e, i, j, l–v).

**Figure 4:**
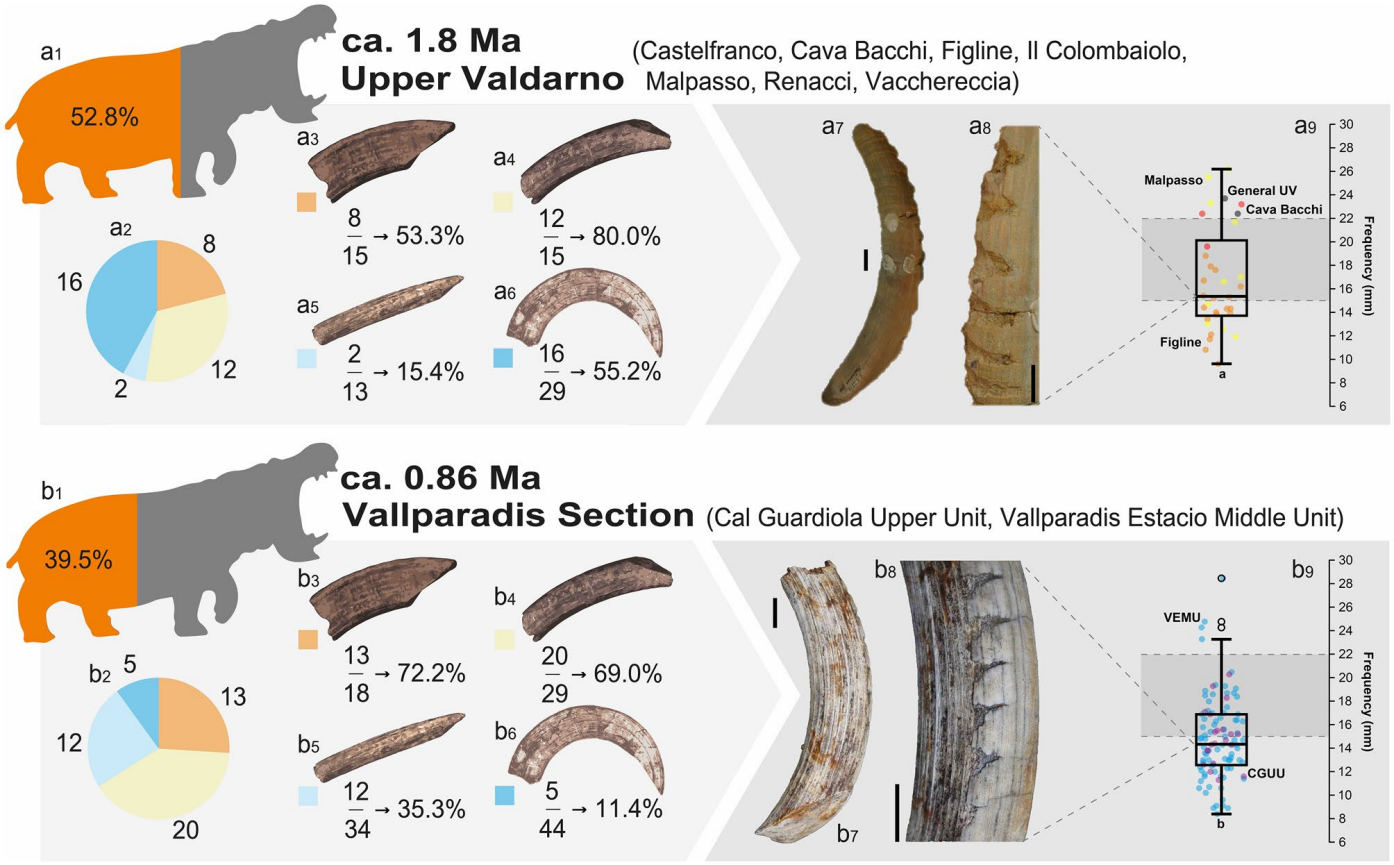
Schematic representation of the main data included in the Dental Enamel Hypoplasia (DEH) analysis of the two most representative samples. (**a**): Upper Valdarno (ca. 1.8 Ma), (**b**): post-Jaramillo Units of Vallparadis Section (ca. 0.86 Ma). a1, b1: percentage of anterior teeth with DEH. a2, b2: teeth-type distribution of the DEH. a3, b3: number of upper incisors with DEH; a4, b4: number of upper canines with DEH; a5, b5: number of lower incisors with DEH; a6, b6: number of lower canines with DEH. a7, 8: reference upper canine with Linear Enamel Hypoplasia (LEH) from Upper Valdarno (NMB Va.2518), b7, 8: reference upper canine with LEH from Vallparadís Section (IPS127141). a9, b9: boxplot of the frequency measurements of isolated episodes of LEH in upper canines, indicating significative differences between samples (Kruskal–Wallis test) with the letters a and b. The area outlined in grey represents the estimated range of annual growth in extant hippos (*Hippopotamus amphibius*). CGUU: Cal Guardiola Upper Unit, VEMU: Vallparadís Estacio Middle Unit. Scale bar equals 20 mm.

In the case of the Upper Valdarno, 52.8% of the observed specimens had enamel pathologies (Fig. 4a1). These pathologies were mainly found in the lower canines (Fig. 4a2; Table S1). On the other hand, 39.5% of the specimens showed enamel pathologies in the post-Jaramillo layers of Vallparadís Section (Fig. 4b1; Table S1). In this sample, pathologies in the upper canines stand out compared to those observed in the lower ones (Fig. 4b2).

The evaluation of the frequency of episodes of LEH in the upper canines (Table S2) resulted in a sample of 7 specimens with 34 measurable oscillations in the Upper Valdarno sample, and 15 specimens with 102 oscillations in the sample from the post-Jaramillo layers of the Vallparadís Section. Oscillations with a mean amplitude of 16.7 mm (Fig. 4a7–9) were found in the Upper Valdarno. In contrast, a mean of 14.8 mm (Fig. 4b7–9) was found in the post-Jaramillo Vallparadís Section. Comparison of the means using the non-parametric Kruskal–Wallis test provided a *p*-value of 0.04463, showing the presence of significant differences between the two samples, which are indicated as subscripts under the error bars in the boxplots shown in Fig. 3a12, b15. Also striking are the extreme cases, with a minimum of 8.4–9.6 mm and a maximum of 26.2–28.5 mm.

## Discussion

### Enamel hypoplasia in Early Pleistocene *Hippopotamus*

Few cases of DEH associated with trauma have been reported, as most pathological specimens show a clear periodicity in the DEH episodes, which allows discarding one-off events in them (see Fig. 3h and Kierdorf and Kahlke^20^). Congenital DEH, which is the pathology least reported in previous studies, shows characteristic typologies that differentiate it from most of the cases observed in the sample studied here^8^. The wide geographical and chronological extension of the sample analysed here depicting these pathologies allows to discard an intoxication aetiology as the cause of the observed DEH^22,23^. Given the characteristic repetitive periodicity of hypoplasia episodes assessed in the pathological anterior dentition, the most likely cause of most pathologies observed in the sample studied here appears to be the repetitive and cyclical lack of nutrients. This interpretation is in line with that inferred in other studies, including those involving DEH in extant hippopotamuses^20,22^ and other ungulates^14,15,24^.

### Hypoplasia frequency in Early Pleistocene *Hippopotamus*

Lower canine growth was studied especially in the extant common hippopotamus, with direct observations of a ratio of 13–14 mm/year in an old (48 years old) female and 28–30 mm/year in a young (8 years old) one^25^. On the other hand, estimates of growth ratios by Uno et al.^26^ provide values of 33.5–74.7 mm/year for the lower canines and 19.4 ± 3.1 mm/year for the upper ones. Harris et al.^27^ considered 30 mm/year as a standard measure of growth for the lower canines, while Souron et al.^28^ highlighted that these values can reach 39.1 mm/year for the lower canines and 30 mm/year for the upper ones. In our case, the estimated frequencies of LEH events in the mean measurements obtained for the upper canines were 16.7 mm and 14.8 mm (Fig. 4a9, b9). The sample analysed comes from individuals of the extinct species *H. antiquus*, which according to estimates made by Palmqvist et al.^21^ could reach a size twice as large as that observed by Nowak and Walker^29^ for the living common hippo (ca. 3200 kg vs. ca. 1500 kg). With the available data, it is difficult to ensure a specific seasonality to the observed episodes of hypoplasia, but everything points to a periodicity of approximately 1–2 episodes per year. Thus, it would be reasonable to consider that these episodes mark the season(s) with most severe weather, which would limit the access of hippopotamuses to their food resources, or with recurrent aridity periods that prevented the access to lacustrine systems with a water table of enough depth.

### Paleoenvironmental inferences

If we focus on the paleoenvironmental context of the samples, the Upper Valdarno specimens (ca. 1.9–1.7 Ma) record apparently drastic episodes for the physiology of individuals of European hippopotamus populations. In fact, in the coeval localities of this basin, seasonal drought events have been hypothesised, especially in the site of Poggio Rosso^3^. These aridity episodes fit with the proposed alternations of cold/warm phases of the European climate, which would in turn results in changes in the distribution of open/closed environments^2,30^. Ultimately, disrupting the access to the vegetation on which the hippopotamuses fed.

Between 1.7 and 1.4 Ma the climate seems to have been associated with more favourable conditions, with the expansion of grasslands^31^. This environment is close to ideal conditions for the maintenance of hippopotamus populations^32^ and might reflect in the absence of DEH in the samples of Venta Micena and Monte Argentario. Nevertheless, we remain cautious in this interpretation, as only two specimens from each site were evaluated. In fact, linear dental hypoplasia was found in specimens of horse *Equus altidens* from Venta Micena, which suggests unfavourable conditions at least for this grazing and fully terrestrial species^33^. Furthermore, previous biogeochemical studies based on the relative abundance of carbon- and nitrogen-stable isotopes retrieved from bone collagen in the late Early Pleistocene site of Venta Micena (Baza Basin, SE Spain) have shown unexpectedly high δ^15^N values in the remains of *H. antiquus* compared to other ungulates recorded at the site. The values were even higher than those measured in the hypercarnivores of the palaeocommunity (e.g., sabertooth cats, hyenas, and wild dogs). These high values of *H. antiquus* in Venta Micena were interpreted as reflecting a diet based mainly on aquatic plants, including the macrophytes and algae that grew in the oligosaline waters of the lacustrine environments of the Baza Basin^21,34^. The feeding behaviour of extant common hippopotamuses is preferentially, and almost exclusively, based on grazing on terrestrial vegetation, and browsing habits have only been reported when grasses are not available^35^. If the interpretation of the Venta Micena isotopic data was confirmed, the feeding habits of the hippopotamuses found at this site would be particularly different from those of the extant populations and might be linked to environmental conditions that limited the proliferation of grazing areas.

In the period 1.4–1.1 Ma, a progressive increase in the amplitude of climate oscillations is recorded being associated with the beginning of the EMPT^2^, which resulted in an appreciable diffusion of Mediterranean vegetation with the intercalation of arid phases and greater seasonality^30^. The sample from this chronological range includes only five pathological specimens (10.2% of the total number of analysed specimens from this chronological range; e.g., Barranco León D; Fig. 1; Table S1). None of the 23 specimens from the Lower Unit of Cal Guardiola (MIS35) show any type of DEH. The palaeoecological reconstructions of Cal Guardiola in these chronologies point to a mixed environment, with the presence of wooded and open areas, leading to the inference of a climate with some permanent humidity^36^ (Fig. 1).

The absence of DEH in the samples dated to ca, 1.0 Ma (MIS31) in the Lower Unit of Vallparadís Estació is noteworthy, although the sample from these localities is very limited (3 specimens). In other samples of the same chronology, such as Untermassfeld or Collecurti, several specimens with pathologies were reported but the prevalence and severity of DEH in them is not excessively high^20^. Palaeoenvironmental reconstructions of these localities, specifically Vallparadís Estació layer EVT12, point to the near absence of DEH in the hippopotamus remains recorded, which coincides with a period of aridity and expansion of grasslands at least in the Mediterranean Europe^36,37^.

During the chronological span comprised between MIS24 to MIS22 (ca. 900 ka), a long and severe glacial phase is recorded^2,38^. The samples dated to ca. 0.90–0.86 Ma include a prevalence of sites with a high incidence of DEH, such as the post-Jaramillo units of Vallparadís Section. In the later site, an increase in humidity and tree cover has been detected, which could limit the expansion of grasslands^37^. It should also be noted that these are the first records of *H. antiquus* after MIS24-22 marking a change in biodiversity of terrestrial communities, so it is possible that plant biodiversity also continued to be affected.

Finally, it is important to make special mention of the significant differences reported in the frequency of the episodes of upper canine hypoplasia between the samples from the Upper Valdarno (ca. 1.9–1.7 Ma) and those from the post-Jaramillo Vallparadís Section (ca. 0.86 Ma) (see Fig. 4a9, b9 and Sect. “Materials and methods”). This phenomenon highlights the presence of a marked climatic temporality and an increase in its frequency that may be in line with postulated changes in seasonality during the late Early Pleistocene around the Mediterranean Basin^39^.

## Material and methods

### Materials

Specimens of the anterior dentition of Early Pleistocene hippopotamuses (*H. antiquus)* from different European sites have been analysed (Table S1; Figs. 1 and S1). These dental elements have been selected for analysis for the continuous growth of the anterior dentition throughout the life of the individuals, which allows the assessment of changes in the type of enamel secretion by ameloblasts at different stages of their life cycle, with a time resolution of few days^25^. Moreover, preliminary observations of cases of dental hypoplasia in the postcanine dentition have not reported any remarkable cases of DEH.

We have selected those samples of European localities dated to the late Early Pleistocene (i.e., Late Villafranchian and Epivillafranchian), with an established presence of hippopotamuses and accessibility of data to the authors (either primary access or through the literature). For a detailed list of the localities studied and all characteristics related to each sample, see Figs. 1, S1 and Table S2.

310 tooth specimens of the anterior dentition were evaluated. The Upper Valdarno sites^40^ and the post-Jaramillo levels of the Vallparadís Section ^41^ were treated as two unique samples in this study, since in both cases the accumulations that make up the samples are very close geographically and minimal differences in the chronology of deposition are accepted.

## Methods

The criteria for the detection of enamel pathologies have been standardised following the typification set out by Goodman and Rose^8^, paying special attention to LEH treated in detailed works such as Dobney and Ervynck^14^. In the specific case of LEH, Fig. 2 shows the different degrees of severity observed in each type of tooth element included in the analysis sample.

Data collection was carried out by direct observation of most samples studied and of those specimens figured in the literature (see Table S1). All cases of dental enamel pathologies found in the anterior dentition were documented in detail, noting the anatomical determination of the element and the typology of the pathologies, taking photographs and scanning the specimens at different 3D resolutions with the most accurate scanner available at each institution (Creality CR—SCAN 01, Precision: 0.1 mm, Resolution: 0.5 mm; Artec Eva, Precision: 0.1 mm, Resolution: 0.2 mm; Artec Space Spider, Precision: 0.05 mm, Resolution: 0.1 mm; Artec Micro, Precision: 0.01 mm, Resolution: 0.029 mm). All data were included in databases for individual observation, detailed analyses, and replicability of the evaluation (Tables S1, S2 and S3).

Sites where the sample was small, or in which the representation of pathologies was null or minimal, were evaluated only qualitatively. The prevalence of hypoplasia in samples from the Upper Valdarno sites and the post-Jaramillo layer of the Vallparadís Section was compared using descriptive statistics and qualitative characteristics (Figs. 3 and 4).

The upper canines were selected for the quantitative comparison of the frequency of LEH episodes in those cases where a sequential pattern was detected, because their growth rates are approximately known in the living *H. amphibius*^25–27^. Frequency measurements were standardized as the measurement of the distance from the middle of one normal enamel attachment phase to the next, measuring from the most proximal part of the canine to the most distal, in 3D models using Meshlab software (Fig. S2, Table S2). For the comparison of the frequency of the LEH episodes between the samples from the Upper Valdarno and the post-Jaramillo Vallparadís Section, box plots of the individual measurements of each episode were made (Fig. 4). A statistical comparison of the differences between the frequencies of the two samples was carried out. Firstly, the normality of the samples was checked using the Shaphiro-Wilk test, observing that both the whole data (W = 0.9455, *p *(normal) = 3.512*10–5) and the samples of the Upper Valdarno (W = 0.9328, *p *(normal) = 0.03769) and the post-Jaramillo Vallparadís Section (W = 0.9492, *p *(normal) = 0.00064) showed departures from normality. Given the absence of normality, the Kruskal–Wallis non-parametric test for comparison of means was performed.

Additionally, direct observations of the sample of *Hippopotamus amphibius* specimens available at the Natural History Museum (London), the Muséum National d'Histoire Naturelle (Paris), the Museo Nacional de Ciencias Naturales (Madrid) and the Museo Anatómico de la Universidad de Valladolid (Valladolid) were made to check the possibility of including a current comparative representation of the incidence and degree of Enamel Hypoplasia. Very few cases of enamel hypoplasia (Fig. S3) were observed in this sample, mostly related to malocclusion (Fig. S3g, i, j), congenital pathologies (Fig. S3m, n) or infections of the entire dental series (Fig. S3p, q, r). Two of the individuals with these pathologies came from zoos (Fig. S3i–f, h), while the other seven were wild or of unknown origin (Fig. S3g, i–r). In none of the cases was the degree of linear enamel hypoplasia as severe as in the *Hippopotamus antiquus* sample, so it was not possible to compare the two samples or to assess quantitatively differences in the frequency of hypoplasia episodes.

### Supplementary Information

Supplementary Information.

## Data Availability

All data generated or analyzed during the study are included in this article and in its Supplementary Information files.
