# Supplementary material for: Increase on environmental seasonality through the European Early Pleistocene inferred from dental enamel hypoplasia
Source: Sci Rep. 2023 Oct 7;13:16941. doi: 10.1038/s41598-023-42936-y (PMC10560273; doi:10.1038/s41598-023-42936-y)
Supplement: Supplementary file 1 — Supplementary Information. [file 41598_2023_42936_MOESM1_ESM.docx]

**Supplementary Information**

**Increase on environmental seasonality through the European Early Pleistocene inferred from dental enamel hypoplasia**

Darío Fidalgo^1^, Antonio Rosas^1^, Saverio Bartolini-Lucenti^2^, Jean-Renaud Boisserie^3^, Luca Pandolfi^4^, Bienvenido Martínez-Navarro^5,6,7^, Paul Palmqvist^8^, Lorenzo Rook^2^, Joan Madurell-Malapeira^2,9,*^

^1^Department of Palaeobiology, Museo Nacional de Ciencias Naturales (CSIC), c/ José Gutiérrez Abascal 2, 28006 Madrid, Spain

^2^Earth Sciences Department, Paleo[Fab]Lab, Università di Firenze, Via G. La Pira 4, 50121 Firenze, Italy

^3^Laboratory Paleontology Evolution Paleoecosystems Paleoprimatology (PALEVOPRIM, UMR CNRS 7262), University of Poitiers, Poitiers, France

^4^Dipartimento di Scienze, Università della Basilicata, Viale dell'Ateneo Lucano, 10, 85100 Potenza, Italy

^5^Institut Català de Paleoecologia Humana i Evolució Social (IPHES-CERCA), Zona Educacional 4, Campus Sescelades URV (Edifici W3), 43007, Tarragona, Spain.

^6^Area de Prehistoria, Universitat Rovira i Virgili (URV), Avda. Catalunya 35, 43002 Tarragona, Spain.

^7^ICREA, Pg. Lluís Companys 23, 08010 Barcelona, Spain.

^8^Departamento de Ecología y Geología, Universidad de Málaga, Campus de Teatinos, 29071 Málaga, Spain

^9^Department of Geology, Faculty of Sciences, Universitat Autònoma de Barcelona, Cerdanyola del Vallès, Spain.

**Table S1**. List of directly studied sites and data taken from the literature with a count on the number, location and type of paleopathology. MNI: Minimum Number of Individuals (estimated with anterior dentition elements).

| **Site** | **Country** | **Chronology** | **Coord.** | **Observation** | **Pathology** | **Total** | **Hypoplasia** | **MNI** | **%MNI hypoplasia** | **U. Incisor** | **U. Canine** | **L. Incisor** | **L. Canine** |
| --- | --- | --- | --- | --- | --- | --- | --- | --- | --- | --- | --- | --- | --- |
| Upper Valdarno (various sites) | Italy | 1.9-1.7 Ma | 43.535076, 11.559804 | This paper | Yes | 72 | 38 | 14 | 64.3 | 8 | 12 | 2 | 16 |
| Venta Micena | Spain | 1.6-1.4 Ma | 37.716250, -2.460861 | This paper | No | 2 | 0 | 1 | 0 | 0 | 0 | 0 | 0 |
| Monte Argentario | Italy | ca. 1.5-1.3 Ma | 42.425471, 11.118390 | This paper | No | 2 | 0 | 2 | 0 | 0 | 0 | 0 | 0 |
| Barranco León | Spain | 1.4-1.3 Ma | 37.724167, -2.450833 | This paper | Yes | 14 | 1 | 3 | 3.33 | 1 | 0 | 0 | 0 |
| Fuente Nueva 3 | Spain | 1.4-1.3 Ma | 37.718167, -2.405472 | This paper | Yes | 10 | 2 | 3 | 33.3 | 1 | 0 | 1 | 0 |
| Cava Santarelli-Madonna della Strada | Italy | 1.3-1.1 Ma | 42.346571, 13.257821 | ^[1]^ | Yes | 1 | 1 | 1 | 100 | 0 | 0 | 0 | 1 |
| Saticula | Italy | ca. 1.2-1.1 Ma | 41.287536, 14.46086 | ^[2]^ | Yes | 1 | 1 | 1 | 100 | 0 | 1 | 0 | 0 |
| Cal Guardiola (Lower Unit) | Spain | 1.2-1.1 Ma (MIS35) | 41.564667, 2.017306 | This paper | No | 23 | 0 | 7 | 0 | 0 | 0 | 0 | 0 |
| Bòvila Ordis | Spain | 1.2-1.1 Ma (MIS35) | 42.141694, 2.752083 | This paper | No | 7 | 0 | 2 | 0 | 0 | 0 | 0 | 0 |
| Vallparadís Estació (Lower Unit) | Spain | 1 Ma (MIS31) | 41.563333, 2.018500 | This paper | No | 3 | 0 | 1 | 0 | 0 | 0 | 0 | 0 |
| Untermassfeld | Germany | 1 Ma (MIS31) | 50.530164, 10.403096 | ^[3]^ | Yes | 2 | 2 | 1 | 100 | 0 | 0 | 0 | 2 |
| Collecurti | Italy | 1 Ma (MIS31) | 42.964540, 12.935889 | This paper | Yes | 10 | 2 | 1 | 100 | 1 | 1 | 0 | 0 |
| Fuensanta del Júcar | Spain | 1 Ma | 39.235611, -2.074083 | ^[4]^ | No | 1 | 0 | 1 | 0 | 0 | 0 | 0 | 0 |
| Barranc de la Boella (El Forn) | Spain | 1.07-0.87 Ma | 41.136167, 1.169806 | This paper | No | 1 | 0 | 1 | 0 | 0 | 0 | 0 | 0 |
| Húescar I | Spain | 0.9 Ma | 37.820139, -2.530250 | This paper | Yes | 5 | 2 | 2 | 50 | 1 | 0 | 0 | 1 |
| Valverde de Calatrava | Spain | 0.9-0.8 Ma | 38.964361, -4.037667 | This paper | No | 2 | 0 | 1 | 0 | 0 | 0 | 0 | 0 |
| Cal Guardiola (Upper Unit) | Spain | 0.86 Ma (MIS21) | 41.564667, 2.017306 | This paper | Yes | 27 | 9 | 6 | 50 | 2 | 5 | 2 | 0 |
| Vallparadís Estació (Middle Unit) | Spain | 0.86 Ma (MIS21) | 41.563333, 2.018500 | This paper | Yes | 102 | 42 | 17 | 47.1 | 11 | 15 | 10 | 5 |
| Incarcal Complex (IN-I, IN-II, IN-V) | Spain | 0.86 Ma (MIS21) | 42.193556, 2.772361 | This paper | No | 12 | 0 | 3 | 0 | 0 | 0 | 0 | 0 |
| Atapuerca (TD8) | Spain | ca. 0.8 Ma | 42.351778, -3.520111 | ^[5]^ | No | 1 | 0 | 1 | 0 | 0 | 0 | 0 | 0 |
| Esparraguera | Spain | Early Pleistocene | 41.543458, 1.873421 | This paper | Yes | 1 | 1 | 1 | 100 | 0 | 0 | 0 | 1 |
| Chambezon | France | Early Pleistocene | 45.371571, 3.243599 | This paper | Yes | 4 | 4 | 2 | 100 | 4 | 0 | 0 | 0 |
| Würzburg-Schalksberg | Germany | ca. 0.8 Ma | 49.816666, 9.95 | ^[6]^ | No | 7 | 0 | 2 | 0 | 0 | 0 | 0 | 0 |
|  |  |  |  |  |  |  |  |  |  |  |  |  |  |

**Table S2.** Prevalence of dental enamel hypoplasia and frequencies among the principal studied sites. U: Upper, L: Lower, I: Incisor, C: Canine.

| **Site** | **Chronology** | **Total** | **Hypoplasia** | **%** | **U. incisor** | **U. canine** | **L. incisor** | **L. canine** | **% UI** | **% UC** | **% LI** | **% LC** |
| --- | --- | --- | --- | --- | --- | --- | --- | --- | --- | --- | --- | --- |
| Upper Valdarno | ca. 1.8 Ma | 72 | 38 | 52,78 | 8 | 12 | 2 | 16 | 53,3333 | 80 | 15,3846 | 55,17 |
| Vallparadís Estació (Middle Unit) | ca. 0.86 Ma | 102 | 42 | 41,18 | 11 | 15 | 10 | 5 | 68,75 | 65,217 | 38,4615 | 15,15 |
| Cal Guardiola (Upper Unit ) | ca. 0.86 Ma | 27 | 9 | 33,33 | 2 | 5 | 2 | 0 | 100 | 83,333 | 25 | 0 |
| Vallparadís Section (post-Jaramillo layers) | ca. 0.86 Ma | 129 | 51 | 39,53 | 13 | 20 | 12 | 5 | 72,2222 | 68,966 | 35,2941 | 11,36 |

**Table S3**. Detailed list of all the specimens studied from the Upper Valdarno and Vallparadís Section the position of the paleopathologies and their LEH frequency. No: number of each hypoplasia line. Position: relative placement of each hypoplasia line to the most proximal hypoplasia line of that specimen.

| **No** | **Region** | **Site** | **Specimen** | **Position** | **Measurement (mm)** |
| --- | --- | --- | --- | --- | --- |
| IGF4059V1 | Upper Valdarno | Upper Valdarno | IGF4059V | 1 | 19,6 |
| IGF4059V2 | Upper Valdarno | Upper Valdarno | IGF4059V | 2 | 23,2 |
| IGF4059V3 | Upper Valdarno | Upper Valdarno | IGF4059V | 3 | 22,4 |
| IGF774a1 | Upper Valdarno | Upper Valdarno | IGF774a | 1 | 12,1 |
| IGF774a2 | Upper Valdarno | Upper Valdarno | IGF774a | 2 | 13,4 |
| IGF774a3 | Upper Valdarno | Upper Valdarno | IGF774a | 3 | 13,8 |
| IGF774a4 | Upper Valdarno | Upper Valdarno | IGF774a | 4 | 14,3 |
| IGF774a5 | Upper Valdarno | Upper Valdarno | IGF774a | 5 | 11,7 |
| IGF7801 | Upper Valdarno | Upper Valdarno | IGF780 | 1 | 13,9 |
| IGF7802 | Upper Valdarno | Upper Valdarno | IGF780 | 2 | 14,4 |
| IGF7803 | Upper Valdarno | Upper Valdarno | IGF780 | 3 | 14 |
| IGF7804 | Upper Valdarno | Upper Valdarno | IGF780 | 4 | 16,7 |
| IGF7831 | Upper Valdarno | Upper Valdarno | IGF783 | 1 | 10,8 |
| IGF7832 | Upper Valdarno | Upper Valdarno | IGF783 | 2 | 14,3 |
| IGF7833 | Upper Valdarno | Upper Valdarno | IGF783 | 3 | 9,6 |
| IGF7834 | Upper Valdarno | Upper Valdarno | IGF783 | 4 | 15,3 |
| IGF7931 | Upper Valdarno | Upper Valdarno | IGF793 | 1 | 16,2 |
| IGF7932 | Upper Valdarno | Upper Valdarno | IGF793 | 2 | 15,4 |
| IGF7933 | Upper Valdarno | Upper Valdarno | IGF793 | 3 | 18,8 |
| IGF7934 | Upper Valdarno | Upper Valdarno | IGF793 | 4 | 15,2 |
| IGF7935 | Upper Valdarno | Upper Valdarno | IGF793 | 5 | 17,6 |
| IGF7936 | Upper Valdarno | Upper Valdarno | IGF793 | 6 | 17,9 |
| IGF7971 | Upper Valdarno | Upper Valdarno | IGF797 | 1 | 23,7 |
| IGF7972 | Upper Valdarno | Upper Valdarno | IGF797 | 2 | 22,4 |
| Va.25181 | Upper Valdarno | Upper Valdarno | Va.2518 | 1 | 11,9 |
| Va.25182 | Upper Valdarno | Upper Valdarno | Va.2518 | 2 | 17 |
| Va.25183 | Upper Valdarno | Upper Valdarno | Va.2518 | 3 | 12,6 |
| Va.25184 | Upper Valdarno | Upper Valdarno | Va.2518 | 4 | 13 |
| Va.25185 | Upper Valdarno | Upper Valdarno | Va.2518 | 5 | 14,7 |
| Va.25186 | Upper Valdarno | Upper Valdarno | Va.2518 | 6 | 16,6 |
| Va.25187 | Upper Valdarno | Upper Valdarno | Va.2518 | 7 | 23,3 |
| Va.25188 | Upper Valdarno | Upper Valdarno | Va.2518 | 8 | 26,2 |
| Va.25189 | Upper Valdarno | Upper Valdarno | Va.2518 | 9 | 25,5 |
| Va.251810 | Upper Valdarno | Upper Valdarno | Va.2518 | 10 | 21,7 |
| IPS1265491 | Vallparadís Section | Vallparadís Estació Middle Unit | IPS126549 | 1 | 11,8 |
| IPS1265492 | Vallparadís Section | Vallparadís Estació Middle Unit | IPS126549 | 2 | 11,4 |
| IPS1265493 | Vallparadís Section | Vallparadís Estació Middle Unit | IPS126549 | 3 | 10,6 |
| IPS1265494 | Vallparadís Section | Vallparadís Estació Middle Unit | IPS126549 | 4 | 10,7 |
| IPS1265495 | Vallparadís Section | Vallparadís Estació Middle Unit | IPS126549 | 5 | 18,5 |
| IPS1265496 | Vallparadís Section | Vallparadís Estació Middle Unit | IPS126549 | 6 | 19,4 |
| IPS1265497 | Vallparadís Section | Vallparadís Estació Middle Unit | IPS126549 | 7 | 18,7 |
| IPS1265498 | Vallparadís Section | Vallparadís Estació Middle Unit | IPS126549 | 8 | 17,2 |
| IPS1266081 | Vallparadís Section | Vallparadís Estació Middle Unit | IPS126608 | 1 | 13,2 |
| IPS1266082 | Vallparadís Section | Vallparadís Estació Middle Unit | IPS126608 | 2 | 14,9 |
| IPS1266083 | Vallparadís Section | Vallparadís Estació Middle Unit | IPS126608 | 3 | 12 |
| IPS1266084 | Vallparadís Section | Vallparadís Estació Middle Unit | IPS126608 | 4 | 11,2 |
| IPS1266085 | Vallparadís Section | Vallparadís Estació Middle Unit | IPS126608 | 5 | 12,8 |
| IPS1266086 | Vallparadís Section | Vallparadís Estació Middle Unit | IPS126608 | 6 | 13,5 |
| IPS1266087 | Vallparadís Section | Vallparadís Estació Middle Unit | IPS126608 | 7 | 13,9 |
| IPS1266088 | Vallparadís Section | Vallparadís Estació Middle Unit | IPS126608 | 8 | 13 |
| IPS1266089 | Vallparadís Section | Vallparadís Estació Middle Unit | IPS126608 | 9 | 16,3 |
| IPS12660810 | Vallparadís Section | Vallparadís Estació Middle Unit | IPS126608 | 10 | 14,8 |
| IPS12660811 | Vallparadís Section | Vallparadís Estació Middle Unit | IPS126608 | 11 | 13 |
| IPS1269591 | Vallparadís Section | Vallparadís Estació Middle Unit | IPS126959 | 1 | 17 |
| IPS1269592 | Vallparadís Section | Vallparadís Estació Middle Unit | IPS126959 | 2 | 16 |
| IPS1269593 | Vallparadís Section | Vallparadís Estació Middle Unit | IPS126959 | 3 | 13,8 |
| IPS1269594 | Vallparadís Section | Vallparadís Estació Middle Unit | IPS126959 | 4 | 13,1 |
| IPS1269595 | Vallparadís Section | Vallparadís Estació Middle Unit | IPS126959 | 5 | 13 |
| IPS1269596 | Vallparadís Section | Vallparadís Estació Middle Unit | IPS126959 | 6 | 18,4 |
| IPS1269597 | Vallparadís Section | Vallparadís Estació Middle Unit | IPS126959 | 7 | 14,7 |
| IPS1269598 | Vallparadís Section | Vallparadís Estació Middle Unit | IPS126959 | 8 | 14,3 |
| IPS1269599 | Vallparadís Section | Vallparadís Estació Middle Unit | IPS126959 | 9 | 14,8 |
| IPS12695910 | Vallparadís Section | Vallparadís Estació Middle Unit | IPS126959 | 10 | 10,2 |
| IPS12695911 | Vallparadís Section | Vallparadís Estació Middle Unit | IPS126959 | 11 | 11,5 |
| IPS1269781 | Vallparadís Section | Vallparadís Estació Middle Unit | IPS126978 | 1 | 13 |
| IPS1269782 | Vallparadís Section | Vallparadís Estació Middle Unit | IPS126978 | 2 | 13 |
| IPS1269783 | Vallparadís Section | Vallparadís Estació Middle Unit | IPS126978 | 3 | 13,5 |
| IPS1269784 | Vallparadís Section | Vallparadís Estació Middle Unit | IPS126978 | 4 | 14,3 |
| IPS1269785 | Vallparadís Section | Vallparadís Estació Middle Unit | IPS126978 | 5 | 13,6 |
| IPS1269786 | Vallparadís Section | Vallparadís Estació Middle Unit | IPS126978 | 6 | 15 |
| IPS1269787 | Vallparadís Section | Vallparadís Estació Middle Unit | IPS126978 | 7 | 12,4 |
| IPS1269981 | Vallparadís Section | Vallparadís Estació Middle Unit | IPS126998 | 1 | 19,1 |
| IPS1269982 | Vallparadís Section | Vallparadís Estació Middle Unit | IPS126998 | 2 | 16,5 |
| IPS1269983 | Vallparadís Section | Vallparadís Estació Middle Unit | IPS126998 | 3 | 15,8 |
| IPS1269984 | Vallparadís Section | Vallparadís Estació Middle Unit | IPS126998 | 4 | 16,3 |
| IPS1269985 | Vallparadís Section | Vallparadís Estació Middle Unit | IPS126998 | 5 | 16,9 |
| IPS1269986 | Vallparadís Section | Vallparadís Estació Middle Unit | IPS126998 | 6 | 19,3 |
| IPS1269987 | Vallparadís Section | Vallparadís Estació Middle Unit | IPS126998 | 7 | 18,9 |
| IPS1269988 | Vallparadís Section | Vallparadís Estació Middle Unit | IPS126998 | 8 | 15,4 |
| IPS1271161 | Vallparadís Section | Vallparadís Estació Middle Unit | IPS127116 | 1 | 11,6 |
| IPS1271162 | Vallparadís Section | Vallparadís Estació Middle Unit | IPS127116 | 2 | 9,1 |
| IPS1271163 | Vallparadís Section | Vallparadís Estació Middle Unit | IPS127116 | 3 | 12,3 |
| IPS1271164 | Vallparadís Section | Vallparadís Estació Middle Unit | IPS127116 | 4 | 9,6 |
| IPS1271165 | Vallparadís Section | Vallparadís Estació Middle Unit | IPS127116 | 5 | 8,9 |
| IPS1271166 | Vallparadís Section | Vallparadís Estació Middle Unit | IPS127116 | 6 | 8,4 |
| IPS1271167 | Vallparadís Section | Vallparadís Estació Middle Unit | IPS127116 | 7 | 12,3 |
| IPS1271168 | Vallparadís Section | Vallparadís Estació Middle Unit | IPS127116 | 8 | 14 |
| IPS1271169 | Vallparadís Section | Vallparadís Estació Middle Unit | IPS127116 | 9 | 15,3 |
| IPS12711610 | Vallparadís Section | Vallparadís Estació Middle Unit | IPS127116 | 10 | 16,4 |
| IPS1271171 | Vallparadís Section | Vallparadís Estació Middle Unit | IPS127117 | 1 | 23,3 |
| IPS1271172 | Vallparadís Section | Vallparadís Estació Middle Unit | IPS127117 | 2 | 28,5 |
| IPS1271381 | Vallparadís Section | Vallparadís Estació Middle Unit | IPS127138 | 1 | 16,6 |
| IPS1271382 | Vallparadís Section | Vallparadís Estació Middle Unit | IPS127138 | 2 | 17,5 |
| IPS1271383 | Vallparadís Section | Vallparadís Estació Middle Unit | IPS127138 | 3 | 18,6 |
| IPS1271384 | Vallparadís Section | Vallparadís Estació Middle Unit | IPS127138 | 4 | 20,5 |
| IPS1271391 | Vallparadís Section | Vallparadís Estació Middle Unit | IPS127139 | 1 | 24,3 |
| IPS1271392 | Vallparadís Section | Vallparadís Estació Middle Unit | IPS127139 | 2 | 24,8 |
| IPS1271411 | Vallparadís Section | Vallparadís Estació Middle Unit | IPS127141 | 1 | 14,3 |
| IPS1271412 | Vallparadís Section | Vallparadís Estació Middle Unit | IPS127141 | 2 | 16,4 |
| IPS1271413 | Vallparadís Section | Vallparadís Estació Middle Unit | IPS127141 | 3 | 17 |
| IPS1271414 | Vallparadís Section | Vallparadís Estació Middle Unit | IPS127141 | 4 | 14,1 |
| IPS1271415 | Vallparadís Section | Vallparadís Estació Middle Unit | IPS127141 | 5 | 16,9 |
| IPS1271416 | Vallparadís Section | Vallparadís Estació Middle Unit | IPS127141 | 6 | 18 |
| IPS1271417 | Vallparadís Section | Vallparadís Estació Middle Unit | IPS127141 | 7 | 18,7 |
| IPS1272591 | Vallparadís Section | Vallparadís Estació Middle Unit | IPS127259 | 1 | 12,1 |
| IPS1272592 | Vallparadís Section | Vallparadís Estació Middle Unit | IPS127259 | 2 | 10,6 |
| IPS1272593 | Vallparadís Section | Vallparadís Estació Middle Unit | IPS127259 | 3 | 12,6 |
| IPS1272594 | Vallparadís Section | Vallparadís Estació Middle Unit | IPS127259 | 4 | 15,6 |
| IPS1272595 | Vallparadís Section | Vallparadís Estació Middle Unit | IPS127259 | 5 | 14,7 |
| IPS1272596 | Vallparadís Section | Vallparadís Estació Middle Unit | IPS127259 | 6 | 15,1 |
| IPS1272597 | Vallparadís Section | Vallparadís Estació Middle Unit | IPS127259 | 7 | 12,8 |
| IPS1272598 | Vallparadís Section | Vallparadís Estació Middle Unit | IPS127259 | 8 | 12,5 |
| IPS1272599 | Vallparadís Section | Vallparadís Estació Middle Unit | IPS127259 | 9 | 11,6 |
| IPS12725910 | Vallparadís Section | Vallparadís Estació Middle Unit | IPS127259 | 10 | 8,9 |
| IPS12725911 | Vallparadís Section | Vallparadís Estació Middle Unit | IPS127259 | 11 | 9,1 |
| IPS135151 | Vallparadís Section | Cal Guardiola Upper Unit | IPS13515 | 1 | 15,2 |
| IPS135152 | Vallparadís Section | Cal Guardiola Upper Unit | IPS13515 | 2 | 18,3 |
| IPS135153 | Vallparadís Section | Cal Guardiola Upper Unit | IPS13515 | 3 | 14,3 |
| IPS135154 | Vallparadís Section | Cal Guardiola Upper Unit | IPS13515 | 4 | 11,6 |
| IPS135155 | Vallparadís Section | Cal Guardiola Upper Unit | IPS13515 | 5 | 14,3 |
| IPS135156 | Vallparadís Section | Cal Guardiola Upper Unit | IPS13515 | 6 | 14,4 |
| IPS135157 | Vallparadís Section | Cal Guardiola Upper Unit | IPS13515 | 7 | 12,7 |
| IPS135158 | Vallparadís Section | Cal Guardiola Upper Unit | IPS13515 | 8 | 16,9 |
| IPS135159 | Vallparadís Section | Cal Guardiola Upper Unit | IPS13515 | 9 | 19,3 |
| IPS135311 | Vallparadís Section | Cal Guardiola Upper Unit | IPS13531 | 1 | 11,3 |
| IPS135312 | Vallparadís Section | Cal Guardiola Upper Unit | IPS13531 | 2 | 12 |
| IPS135313 | Vallparadís Section | Cal Guardiola Upper Unit | IPS13531 | 3 | 13,8 |
| IPS135314 | Vallparadís Section | Cal Guardiola Upper Unit | IPS13531 | 4 | 12,6 |
| IPS135315 | Vallparadís Section | Cal Guardiola Upper Unit | IPS13531 | 5 | 15,2 |
| IPS135316 | Vallparadís Section | Cal Guardiola Upper Unit | IPS13531 | 6 | 13,8 |
| IPS136551 | Vallparadís Section | Cal Guardiola Upper Unit | IPS13655 | 1 | 15,4 |
| IPS136552 | Vallparadís Section | Cal Guardiola Upper Unit | IPS13655 | 2 | 15,6 |
| IPS136553 | Vallparadís Section | Cal Guardiola Upper Unit | IPS13655 | 3 | 14,7 |
| IPS136554 | Vallparadís Section | Cal Guardiola Upper Unit | IPS13655 | 4 | 15,5 |
| IPS136555 | Vallparadís Section | Cal Guardiola Upper Unit | IPS13655 | 5 | 17,1 |
| IPS230611 | Vallparadís Section | Cal Guardiola Upper Unit | IPS23061 | 1 | 20,3 |


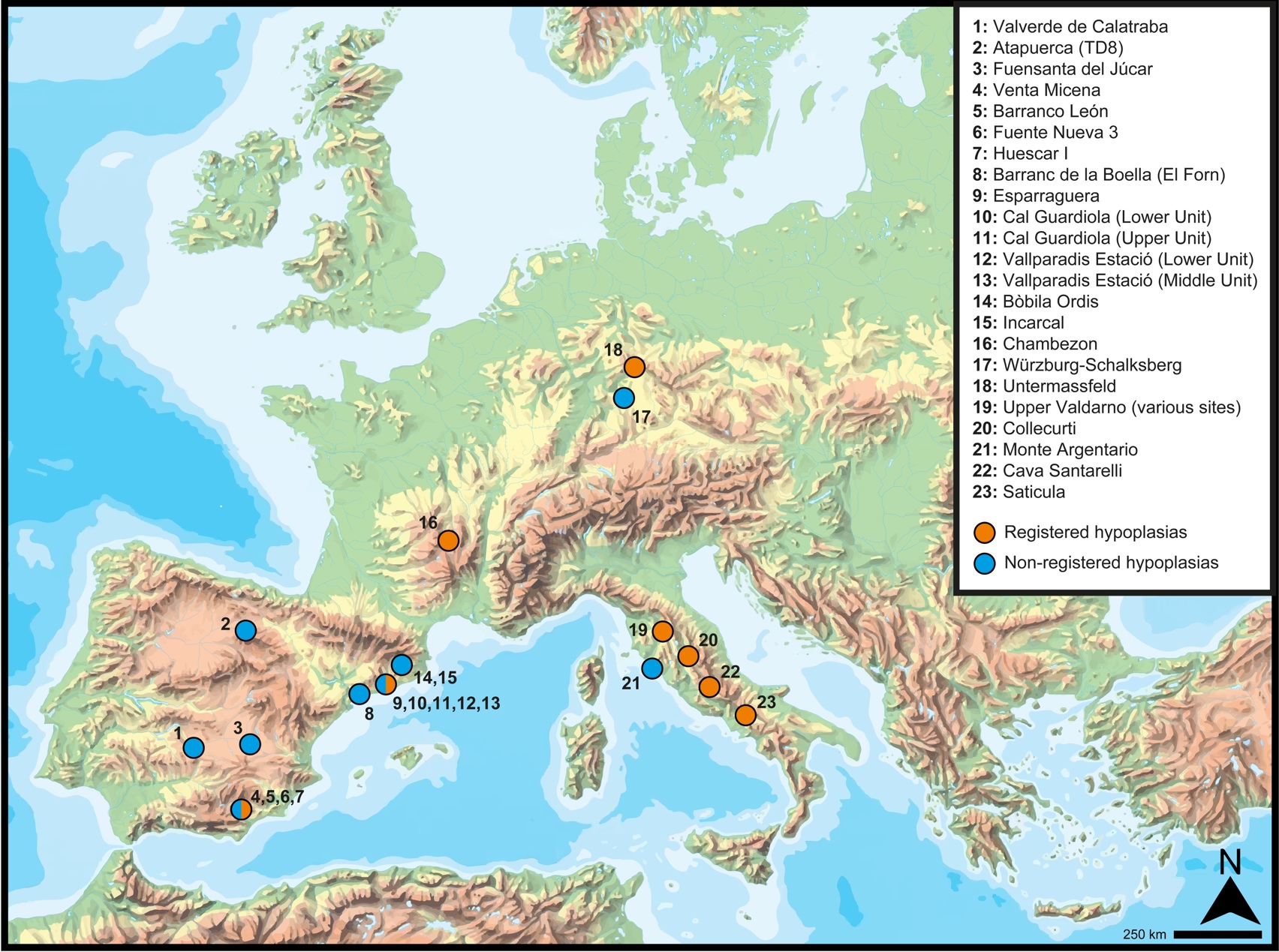


**Figure S1.** Geographical location of the specimens included in this study. Sites with documented hypoplasia are marked in orange; in blue, sites where it was not detected.


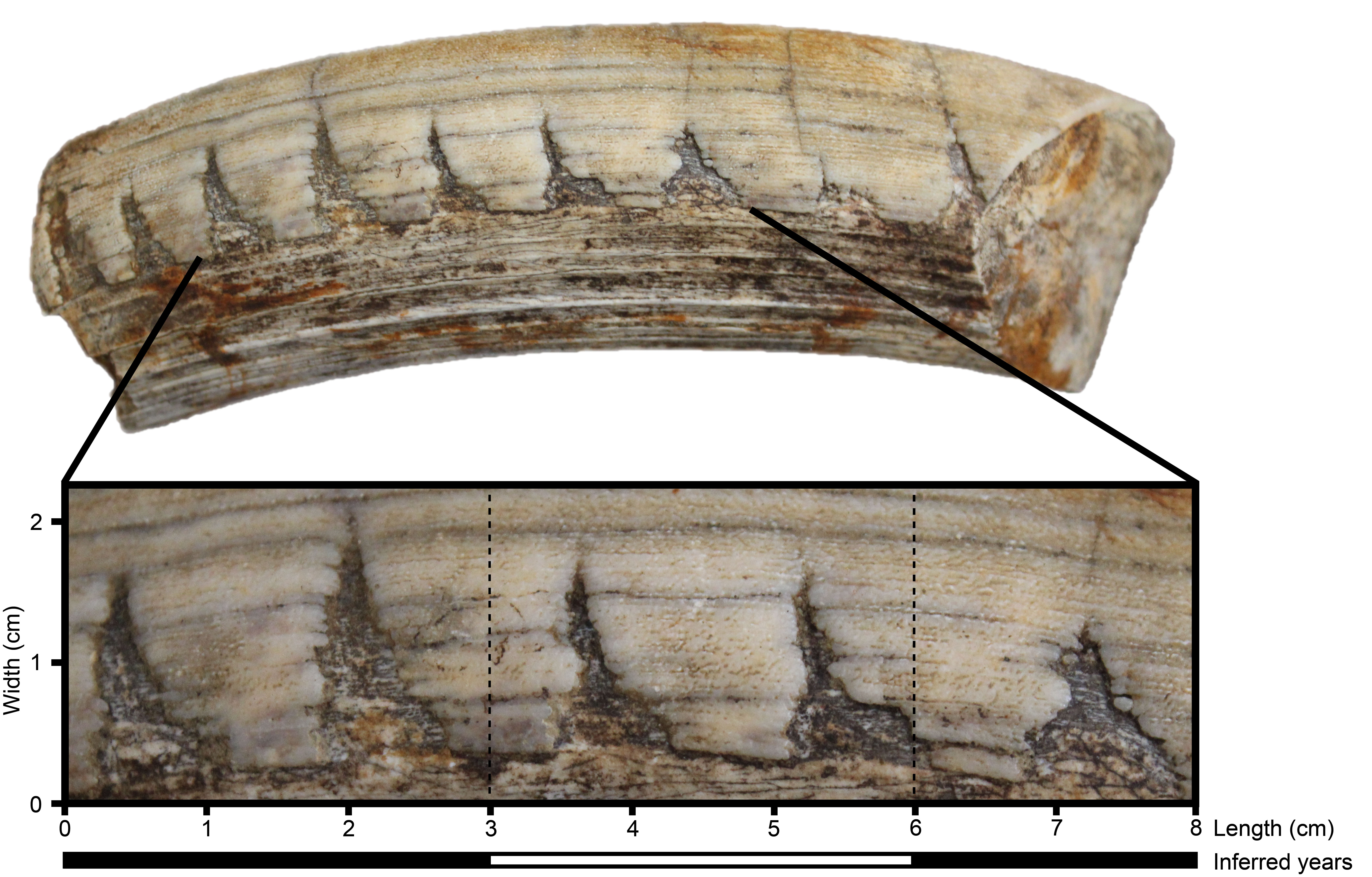


**Figure S2.** Schematic representation of the methodology used in the frequency measurement of the Lineal Enamel Hypoplasia events in the upper canines. Specimen: IPS127141. Three inferred years estimated, in the white and black bar at the bottom, following Harris et al.^[7]^. For more information, see Discussion.

**Figure S3.** Sample of specimens of *Hippopotamus amphibius* with pathologies in the anterior dentition. a-f) specimen MNCN_M22039 (Museo Nacional de Ciencias Naturales), skull and jaw of a female from a zoo in Spain. a) Upper left canine with LEH, b) upper left second incisor with LEH, c) upper left first incisor with LEH, d) lower first incisor with LEH, e) lower left second incisor with PEH and LEH, f) lower left canine with LEH. g and i) Specimen ZD_1984_519 (Natural History Museum UK: NHMUK), jaw and upper canine of an individual from Kenya with an extreme case of malocclusion and overgrowth of the right upper and lower canines with LEH. H) Specimen UVa_6134 (Museo Anatómico de la Universidad de Valladolid), skull and jaw of a female from the zoo Safari Madrid in Spain with LEH in the lower canines. j) Specimen 2017_1178 (Muséum National d'Histoire Naturelle: MNHN), lower right canine with malocclusion, overgrowth and LEH. k) Specimen AC_1895_431 (MNHN), upper right canine of an individual from Republic of Guinea with malocclusion and LEH. l) Specimen ZD_1863_11_12_51 (NHMUK), upper left second incisor of a male from South Africa with PEH. m) Specimen ZE_1958_8_14_4 (NHMUK), upper left first incisor of a male with malformation. n) Specimen 1882_293 (NHMUK), upper canine with extreme malformation. o) Specimen ZE_1962_2_14_10 (NHMUK), upper right second incisor of an individual from Zimbawe with LEH-Plane Enamel Hypoplasia. p, q and r) Specimen AC_1897_33 (MNHN), lower right canine, upper right first incisor and upper left canine of a female individual from Egypt with LEH and infections in the entire dental series. LEH: Linear Enamel Hypoplasia; PEH: Pit Enamel Hypoplasia.

**References**

1. Mancini, M. *et al.* Coupling basin infill history and mammal biochronology in a Pleistocene intramontane basin: The case of western L’Aquila Basin (central Apennines, Italy). *Quat. Int.* **267**, 62-77 (2012).

2. Cicia, V. *et al.* Nuove segnalazioni di *Hippopotamus* Linnaeus, 1758 nel Pleistocene inferiore e medio dell'Italia peninsulare. *XVI Edizione delle Giornate di Paleontologia*, Faenza, 25-27 May (2016).

3. Kierdorf, U., & Kahlke, R. D. (2022). Pathological findings on remains of hippopotamids from the Early Pleistocene site of Untermassfeld in *The Pleistocene of Untermassfeld near Meiningen (Thüringen, Germany)* (ed. Kahlke, R. D.) 1251-1272 (Monographien des RGZM Band 40, 4, 2020).

4. Mazo, A. V., Perez-Gonzalez, A., Aguirre, E. Las faunas pleistocenas de Fuensanta del Jucar y El Provencio y su significado en la evolución del Cuaternario de la Llanura manchega. *Bol. Geol. Min.* **101(3)**, 404–418 (1990).

5. van der Made, J., Rosell, J., Blasco, R. Faunas from Atapuerca at the Early–Middle Pleistocene limit: The ungulates from level TD8 in the context of climatic change. *Quat. Int.* **433**, 296-346 (2017).

6. Kahlke, R. D. Die unterpleistozänen Hippopotamus-Reste von Würzburg-Schalksberg. *Quartär–Internationales Jahrbuch zur Erforschung des Eiszeitalters und der Steinzeit*, 67-94 (1989).

7. Harris, J. M., Cerling, T. E., Leakey, M. G., & Passey, B. H. Stable isotope ecology of fossil hippopotamids from the Lake Turkana Basin of East Africa. *J. Zool.* **275(3)**, 323-331 (2008).
